# RST2G: Residual-Guided Spatiotemporal Transformer Graph Fusion Enhancement for Breast Cancer Segmentation in DCE-MRI

**DOI:** 10.34133/cbsystems.0502

**Published:** 2026-03-23

**Authors:** Shaoli Xie, Lulu Xu, Chenyi Lei, Jinxiang Wang, Jason Wang, Zhibin Wang, Yiran Sun, Danyi Li, Fangfang Li, Rubing Lin, Hongwei Yang, Yang Xiao, Tianxu Lv, Yixuan Huang, Lingmi Hou, Junyan Li, Maoshan Chen

**Affiliations:** ^1^ Department of Thyroid and Breast Surgery, Affiliated Hospital of North Sichuan Medical College, Nanchong, Sichuan 637000, China.; ^2^ Tsinghua Shenzhen International Graduate School, Tsinghua University, Shenzhen 518055, China.; ^3^ Department of Urology, Kidney and Urology Center, Pelvic Floor Disorders Center, The Seventh Affiliated Hospital, Sun Yat-sen University, Shenzhen, Guangdong 518107, China.; ^4^ Department of Clinical Medicine, North Sichuan Medical College, Nanchong, Sichuan 637000, China.; ^5^ Department of Surgical Anesthesia, Suining Central Hospital, Suining, Sichuan 629000, China.; ^6^ Department of Orthopedics, Shenzhen Children’s Hospital, Shenzhen, Guangdong 518000, China.; ^7^ Department of Breast and Thyroid Surgery, Suining Central Hospital, Suining, Sichuan 629000, China.; ^8^ Department of Breast Surgery, Sichuan Clinical Research Center for Cancer, Sichuan Cancer Hospital & Institute, Sichuan Cancer Center, Affiliated Cancer Hospital of University of Electronic Science and Technology of China, Chengdu 610041, China.; ^9^ Department of Thyroid and Breast Surgery, Chengdu Fifth People’s Hospital, The Fifth People’s Hospital Affiliated to Chengdu University of Traditional Chinese Medicine, Chengdu 611130, Sichuan, China.

## Abstract

Accurate segmentation of breast tumors in dynamic contrast-enhanced magnetic resonance imaging (DCE-MRI) is essential for effective diagnosis, treatment planning, and monitoring of breast cancer. However, the high heterogeneity of tumor appearance and the complex spatiotemporal dynamics of contrast enhancement present critical challenges for existing segmentation methods. In this study, we propose a novel residual-guided spatiotemporal transformer with graph fusion enhancement (RST2G) framework for precise breast tumor segmentation in DCE-MRI. RST2G explicitly leverages pre-contrast MRI, post-contrast MRI, and their residual differences to capture rich inter-temporal kinetic information. Specifically, RST2G employs a weight-sharing hybrid encoder that combines convolutional neural networks and vision transformers to extract local and global features, followed by a residual-guided multi-scale refinement module to enhance feature discriminability. To effectively model spatial and temporal contextual dependencies, we construct modality-specific graphs and apply inter-slice and inter-temporal attention mechanisms for spatiotemporal graph enhancement. Extensive experiments on 2 publicly available breast DCE-MRI datasets demonstrate that RST2G significantly outperforms state-of-the-art 2-dimensional (2D), 3D, and 4D segmentation methods. Given its effectiveness in capturing complex spatiotemporal tumor characteristics for cancer annotation, RST2G has the potential to improve clinical breast cancer treatment.

## Introduction

Breast cancer remains one of the most prevalent and deadly malignancies affecting women worldwide [1]. Early and accurate detection of breast tumors is crucial for improving patient prognosis and guiding effective treatment strategies [2]. Precise delineation of tumor boundaries in medical images plays a vital role in diagnosis, treatment planning, and monitoring therapeutic response [3]. However, manual annotation of breast tumors in medical images is labor-intensive and time-consuming, requires expert knowledge, and is prone to inter- and intra-observer variability, limiting its scalability and consistency in clinical practice.

Dynamic contrast-enhanced magnetic resonance imaging (DCE-MRI) has emerged as a powerful tool for breast cancer detection and characterization [4]. It provides both morphological and functional information by capturing the kinetic behavior of contrast agents in tissue [5]. The high sensitivity of DCE-MRI makes it particularly valuable for identifying subtle lesions and assessing tumor angiogenesis [6]. Moreover, DCE-MRI offers rich spatiotemporal information that enables more accurate characterization of tumor heterogeneity and vascular properties compared to static imaging techniques [7].

In recent years, deep learning methods have rapidly advanced [8] and have been extensively applied in medical image analysis [9,10], including breast tumor segmentation in DCE-MRI [5]. Traditional machine learning approaches often rely on handcrafted features, which may not fully capture the complex patterns in medical images. In contrast, deep learning architectures, particularly convolutional neural networks (CNNs) [11], have demonstrated superior performance by automatically learning hierarchical features from large datasets. These networks effectively capture spatial and appearance features of tumors, enabling more accurate and efficient segmentation than conventional methods. Furthermore, integrating temporal information and advanced architectures such as transformers [12,13] has enhanced the ability to model complex spatiotemporal dependencies inherent in DCE-MRI sequences, facilitating more robust and precise tumor delineation.

Despite these advances, several challenges remain. First, the high heterogeneity of breast tumors—including variable sizes, shapes, and internal textures—poses significant difficulties for accurate segmentation [14]. Second, although dynamic enhancement patterns contain critical diagnostic cues, many approaches lack effective mechanisms to deeply mine and represent this information with sufficient discriminative power. Therefore, developing an effective method that captures the complex spatiotemporal characteristics of breast tumors is highly promising.

To address these challenges, we propose a residual-guided spatiotemporal transformer with graph fusion enhancement (RST2G) to fully exploit spatiotemporal priors for accurate breast cancer segmentation in DCE-MRI. Specifically, RST2G explicitly incorporates pre-contrast MRI, post-contrast MRI, and their residuals as inputs. A weight-sharing convolutional Transformer Encoder (CFormerEncoder) is devised to capture both local and global features from each input branch. We also design a residual-guided multi-scale refinement (MSR) module to enhance the learned representations. Furthermore, we construct pre-contrast, post-contrast, and residual graphs based on the refined features. Using these graphs, we deploy inter-slice attention (ISA) and inter-temporal attention (ITA) mechanisms to capture spatiotemporal contextual information. Finally, the spatiotemporal graph is projected and fed into a CDecoder to generate the final voxel-level segmentation output. Particularly, incorporating pre-contrast MRI, post-contrast MRI, and their residual difference images allows the model to access complementary information reflecting both structural morphology and dynamic contrast enhancement patterns. Pre-contrast images provide baseline anatomical context; post-contrast images capture functional changes following contrast agent uptake; and residual images explicitly highlight areas of dynamic enhancement critical for identifying tumor regions. Extensive experiments demonstrate that RST2G outperforms recent state-of-the-art 2-dimensional (2D), 3D, and 4D approaches, highlighting its potential to advance breast cancer segmentation in DCE-MRI.

## Results

### The RST2G algorithm

**Fig. 1.**
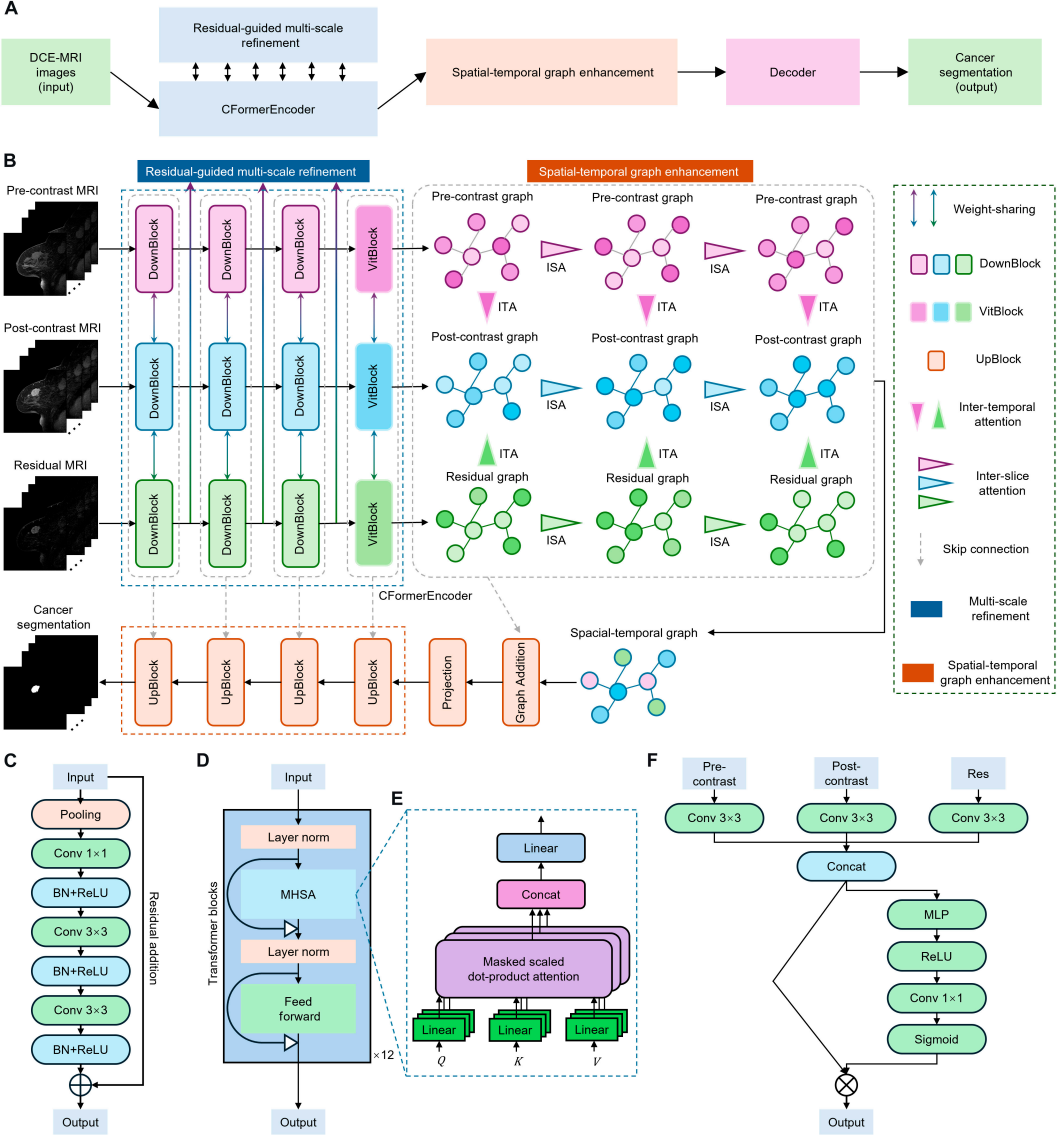
(A) A schematic that describes the module flow of the proposed RST2G. (B) Overall architecture of the proposed RST2G. (C) Detailed structure of DownBlocks. (D) Detailed structure of VitBlocks. (E) Computational process of the MHSA mechanism. (F) Computational process of residual-guided MSR.

The RST2G model is a novel framework (Fig. 1) specifically designed for accurate breast cancer segmentation in DCE-MRI. While it builds upon well-established building blocks—including CNNs, vision transformers (ViTs), and graph attention mechanisms—its originality lies in the synergistic integration of these components, tailored to effectively capture the complex spatial and temporal patterns present in breast MRI data.

**Fig. 2.**
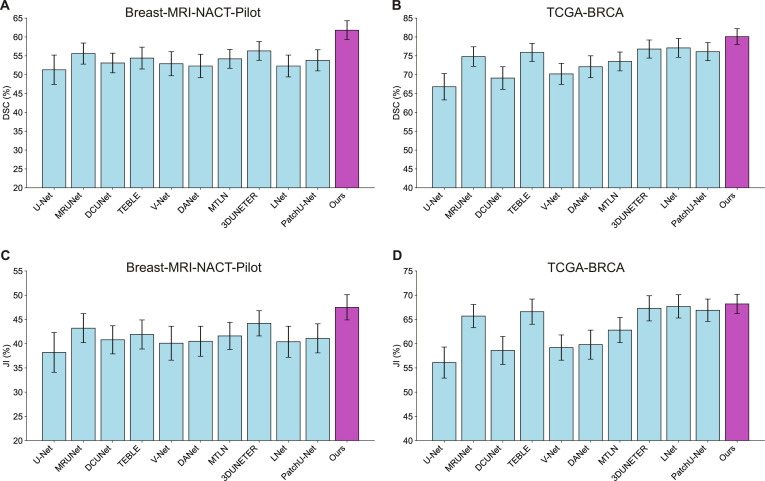
Quantitative comparison between 2D, 3D, 4D competing approaches and the proposed method in 2 different datasets. (A and B) Comparison of DSC metric on Breast-MRI-NACT-Pilot (A) and TCGA-BRCA (B) datasets. (C and D) Comparison of JI metric on Breast-MRI-NACT-Pilot (C) and TCGA-BRCA (D) datasets (mean ± SD).

RST2G leverages residual learning and multi-modal inputs to exploit complementary information that enhances tumor localization. At the input stage, it simultaneously processes pre-contrast MRI, post-contrast MRI, and the residual difference images computed between them. The residual images emphasize subtle intensity changes induced by contrast agents, which are critical for highlighting tumor regions but have been underutilized in prior works with this combined approach.

The core feature extractor, CFormerEncoder, is a hybrid architecture combining CNN layers with Transformer blocks. The CNN layers capture fine-grained local details, while the Transformer components model long-range dependencies and global contextual relationships through self-attention mechanisms. This combination allows the encoder to produce rich and discriminative features that represent complex tissue structures more comprehensively than either architecture alone.

To further refine these features, an MSR module is introduced. The MSR module fuses multi-modal features at different scales, effectively leveraging complementary information across the pre-contrast, post-contrast, and residual modalities. Additionally, spatial attention within this module adaptively recalibrates fused features, emphasizing regions most relevant for tumor segmentation and improving feature discriminability.

A key innovation of RST2G is its graph-based spatiotemporal modeling. To capture contextual relationships not only within individual slices but also across time points and imaging phases, 2 attention mechanisms are designed: ISA and ITA. ISA models spatial dependencies among adjacent slices by treating each spatial location as a graph node and applying graph attention, capturing spatial continuity of breast tissue. Batch-wise attention further enhances the inter-slice feature interactions, strengthening spatial representation. ITA focuses on temporal and modality-wise feature fusion by integrating pre-contrast, post-contrast, and residual features channel-wise via a multi-layer perceptron (MLP) followed by spatial attention, allowing the model to leverage temporal dynamics crucial for delineating tumor boundaries over time.

Finally, the enhanced features are decoded by the CDecoder network, which progressively upsamples and refines feature maps to generate high-resolution segmentation masks. The model is supervised using a hybrid loss function that combines Dice loss, binary cross-entropy (BCE) loss, and boundary loss, ensuring accurate overlap with the ground truth and precise boundary delineations—both vital for clinical application.

In summary, while RST2G employs established CNN, Transformer, and graph attention modules, its novelty derives from the carefully crafted architecture that integrates these elements with residual image inputs, multi-scale multi-modal fusion, and novel spatiotemporal graph attention mechanisms. This comprehensive design enables RST2G to capture complex spatiotemporal patterns in DCE-MRI data more effectively than prior works, leading to improved breast cancer segmentation performance as validated by our extensive experiments.

### Datasets and implementation details

In this study, we evaluate the proposed method using 2 publicly accessible breast cancer datasets [15] that differ in their imaging protocols. The first dataset, Breast-MRI-NACT-Pilot [16], comprises longitudinal DCE-MRI scans from 64 patients undergoing neoadjuvant chemotherapy (NACT) for invasive breast cancer. For each patient, at least 3 time points were acquired during the contrast-enhanced MRI protocol: a pre-contrast scan followed by 2 consecutive post-contrast scans. The contrast agent gadopentetate dimeglumine was administered at a dose of 0.1 mmol/kg body weight, followed by a 10-ml saline flush, with injection synchronized to the start of the early phase acquisition. The post-contrast imaging was performed at 2.5 and 7.5 min after contrast injection using standard *k*-space sampling. Each breast MR volume consists of 60 slices, each with a resolution of 256 × 256 pixels. Tumor annotations are provided in this dataset. The second dataset, The Cancer Genome Atlas Breast Invasive Carcinoma Collection (TCGA-BRCA) [17], contains longitudinal DCE-MRI data from 139 participants. These breast MRIs were acquired using GE 1.5 Tesla scanners (GE Medical Systems, Milwaukee, WI, USA), including one pre-contrast image and between 3 and 5 post-contrast images following contrast agent injection. The in-plane resolution ranges from 0.53 to 0.85 mm, with slice thickness varying between 2 and 3 mm. Each MR volume comprises 60 slices, each sized 256 × 256 pixels. Tumor annotations are provided in this dataset. In addition, we used the publicly accessible breast cancer dataset [18] for external testing, which included 100 cases obtained from Yunnan Cancer Hospital.

We validate the effectiveness of the proposed method using several representative metrics, including Dice similarity coefficient (DSC), Jaccard index (JI), and relative volume difference (RVD). DSC and JI measure the overlap between the predicted and ground truth regions, with higher values indicating better segmentation accuracy. The RVD quantifies the relative difference in volume between the prediction and ground truth, where values closer to zero indicate more accurate volume estimation.

The proposed model was developed using the PyTorch framework and trained on 2 NVIDIA Tesla V100 graphics processing units (GPUs) to accelerate computation. Optimization was performed using the Adam optimizer [19] with an initial learning rate of 10^−3^ and a weight decay of 10^−4^. The batch size was set to 4. The hyperparameters λ and β were empirically set to 0.5 and 0.2, respectively, to balance the loss components. To ensure a fair comparison, all models, including ours and the baselines, were randomly initialized without pretraining on external datasets.

To evaluate the effectiveness of the proposed method, we compared it against several state-of-the-art segmentation approaches spanning 2D, 3D, and 4D frameworks. The 2D methods include U-Net [20], MultiResUNet [21], DCUNet [22], and TEBLS [23], while the 3D methods comprise V-Net [24], DANet [25], MTLN [26], and 3DUNETER [27]. The 4D approaches evaluated are LNet [28] and 3D Patch U-Net [29]. Specifically, U-Net is a widely adopted encoder–decoder architecture with skip connections that facilitate precise localization and context integration. MultiResUNet enhances U-Net by incorporating multi-resolution analysis to capture features at different scales, whereas DCUNet introduces densely connected convolutional blocks to improve feature propagation and gradient flow. V-Net extends U-Net to volumetric data using 3D convolutions for effective volumetric segmentation. DANet integrates dual attention mechanisms to capture spatial and channel-wise dependencies, improving segmentation accuracy. MTLN employs multi-task learning to jointly optimize related tasks, enhancing robustness. LNet and 3D Patch U-Net exploit temporal and spatial information in 4D data, with LNet emphasizing longitudinal consistency and 3D Patch U-Net utilizing patch-based processing to capture local details effectively.

### Quantitative comparison with state-of-the-art methods

We quantitatively compared our method with these baselines on 2 publicly available breast cancer datasets: Breast-MRI-NACT-Pilot and TCGA-BRCA. The results, summarized in Tables 1 and 2, demonstrate the superior performance of our approach across all evaluation metrics, including DSC, JI, and RVD. On the Breast-MRI-NACT-Pilot dataset (Table 1), our method achieved a DSC of 61.8%, significantly outperforming all competing methods. Among the 2D approaches, MultiResUNet attained the highest DSC of 55.6%, followed by DCUNet and U-Net. The 3D methods—V-Net, DANet, and MTLN—showed comparable performance to the 2D models, with MTLN achieving the best DSC of 54.2% within this group. The 4D methods, LNet and 3D Patch U-Net, improved upon the 2D and 3D baselines by leveraging temporal information, reaching DSCs of 52.3% and 53.8%, respectively. Notably, our approach surpassed these by a substantial margin, underscoring the effectiveness of incorporating spatiotemporal features for longitudinal breast tumor segmentation. Regarding other metrics, our method also attained the highest JI of 47.5%, exceeding the best baseline, MultiResUNet, by over 4 percentage points. Furthermore, our model minimized the RVD metric to 1.8 voxels, indicating more precise tumor volume delineation compared to other methods.

**Table 1.** 2D, 3D, and 4D segmentation performance of various approaches on the Breast-MRI-NACT-Pilot dataset (mean ± SD)

Method	Modality	DSC/%	JI/%	RVD/voxel
U-Net	2D	51.3 ± 3.9	38.2 ± 4.1	3.3 ± 1.7
MultiResUNet	2D	55.6 ± 2.8	43.2 ± 3.0	2.4 ± 1.0
DCUNet	2D	53.1 ± 2.6	40.8 ± 2.9	2.1 ± 1.0
TEBLS	2D	54.4 ± 2.9	41.9 ± 3.0	2.0 ± 1.0
V-Net	3D	52.9 ± 3.2	40.1 ± 3.5	3.4 ± 1.0
DANet	3D	52.3 ± 3.1	40.5 ± 3.1	6.3 ± 1.6
MTLN	3D	54.2 ± 2.5	41.6 ± 2.8	2.1 ± 1.3
3DUNETER	3D	56.3 ± 2.5	44.2 ± 2.6	2.0 ± 1.3
LNet	4D	52.3 ± 2.9	40.4 ± 3.2	2.7 ± 1.3
3D Patch U-Net	4D	53.8 ± 2.8	41.1 ± 3.0	2.2 ± 1.4
Ours	4D	61.8 ± 2.5	47.5 ± 2.6	1.8 ± 1.1

**Table 2.** 2D, 3D, and 4D segmentation performance of various approaches on the TCGA-BRCA dataset (mean ± SD)

Method	Modality	DSC/%	JI/%	RVD/voxel
U-Net	2D	66.8 ± 3.5	56.1 ± 3.2	2.0 ± 1.3
MultiResUNet	2D	74.8 ± 2.6	65.7 ± 2.4	1.7 ± 0.9
DCUNet	2D	69.1 ± 3.0	58.6 ± 2.9	2.0 ± 1.1
TEBLS	2D	75.9 ± 2.4	66.6 ± 2.6	1.6 ± 1.1
V-Net	3D	70.2 ± 2.8	59.2 ± 2.6	1.9 ± 1.1
DANet	3D	72.1 ± 2.9	59.8 ± 3.0	1.9 ± 1.0
MTLN	3D	73.5 ± 2.5	62.8 ± 2.6	1.8 ± 1.1
3DUNETER	3D	76.8 ± 2.4	67.3 ± 2.6	1.6 ± 1.0
LNet	4D	77.1 ± 2.5	67.7 ± 2.4	1.6 ± 0.9
3D Patch U-Net	4D	76.1 ± 2.4	66.9 ± 2.3	1.6 ± 1.0
Ours	4D	80.1 ± 2.1	68.2 ± 2.0	1.5 ± 0.8

Similar trends were observed on the TCGA-BRCA dataset (Table 2), where our method achieved a DSC of 80.1%, outperforming the second-best method, LNet, by nearly 3 percentage points. The 2D models generally performed better on this dataset compared to Breast-MRI-NACT-Pilot, with MultiResUNet reaching a DSC of 74.8%. The 3D approaches also showed improved results, with MTLN achieving a DSC of 73.5%. The 4D methods, LNet and 3D Patch U-Net, further enhanced segmentation accuracy by exploiting temporal dynamics, achieving DSCs of 77.1% and 76.1%, respectively. The superior performance of our method across all metrics highlights its robustness and generalizability across datasets with varying imaging protocols. Overall, these quantitative results confirm that the proposed method effectively integrates spatial and temporal information, leading to more accurate and reliable breast tumor segmentation in longitudinal DCE-MRI scans.

### Qualitative comparison with state-of-the-art methods

**Fig. 3.**
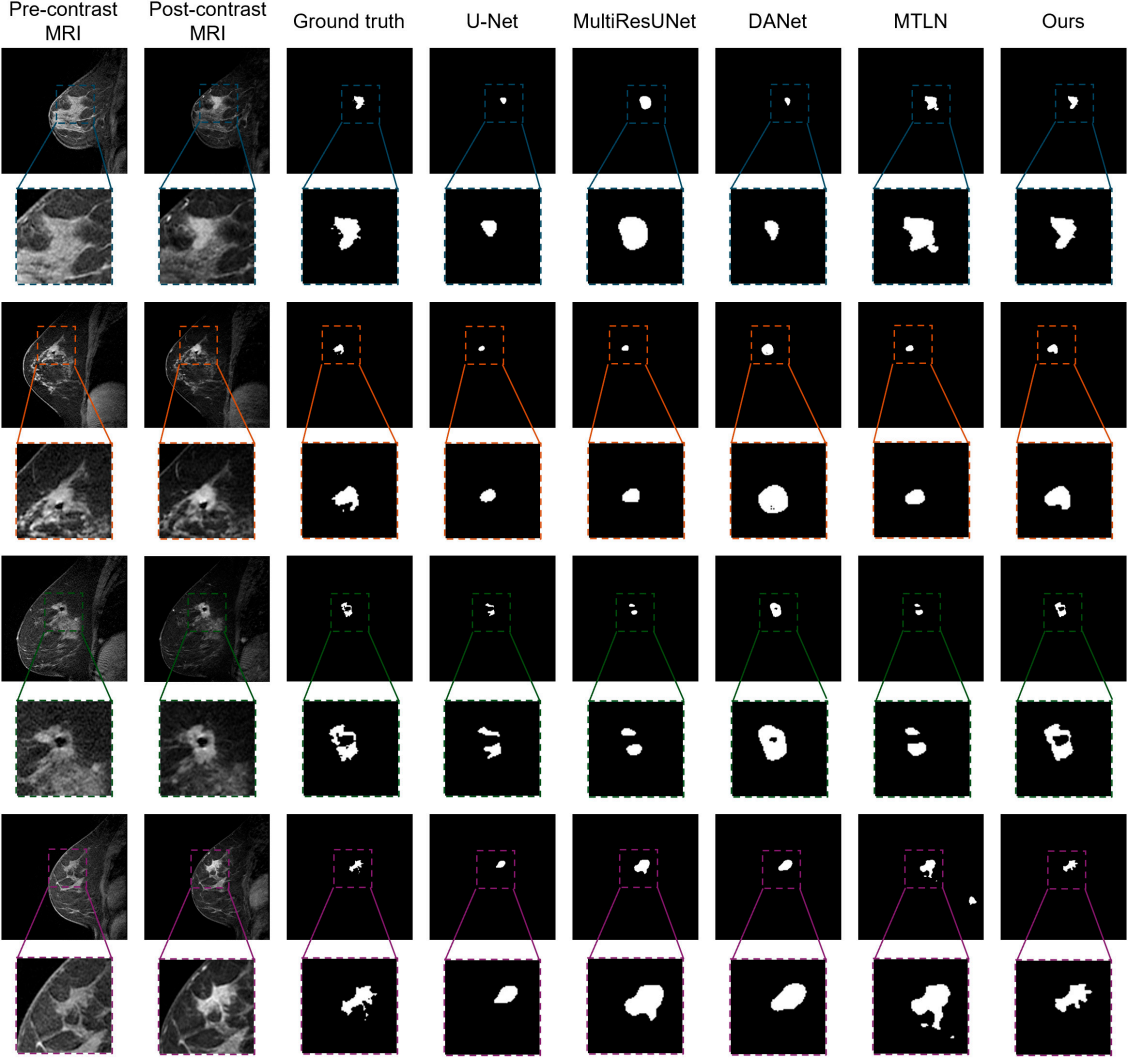
Visual comparison between 2D, 3D, and our proposed 4D segmentation methods in annotating breast tumors.

**Fig. 4.**
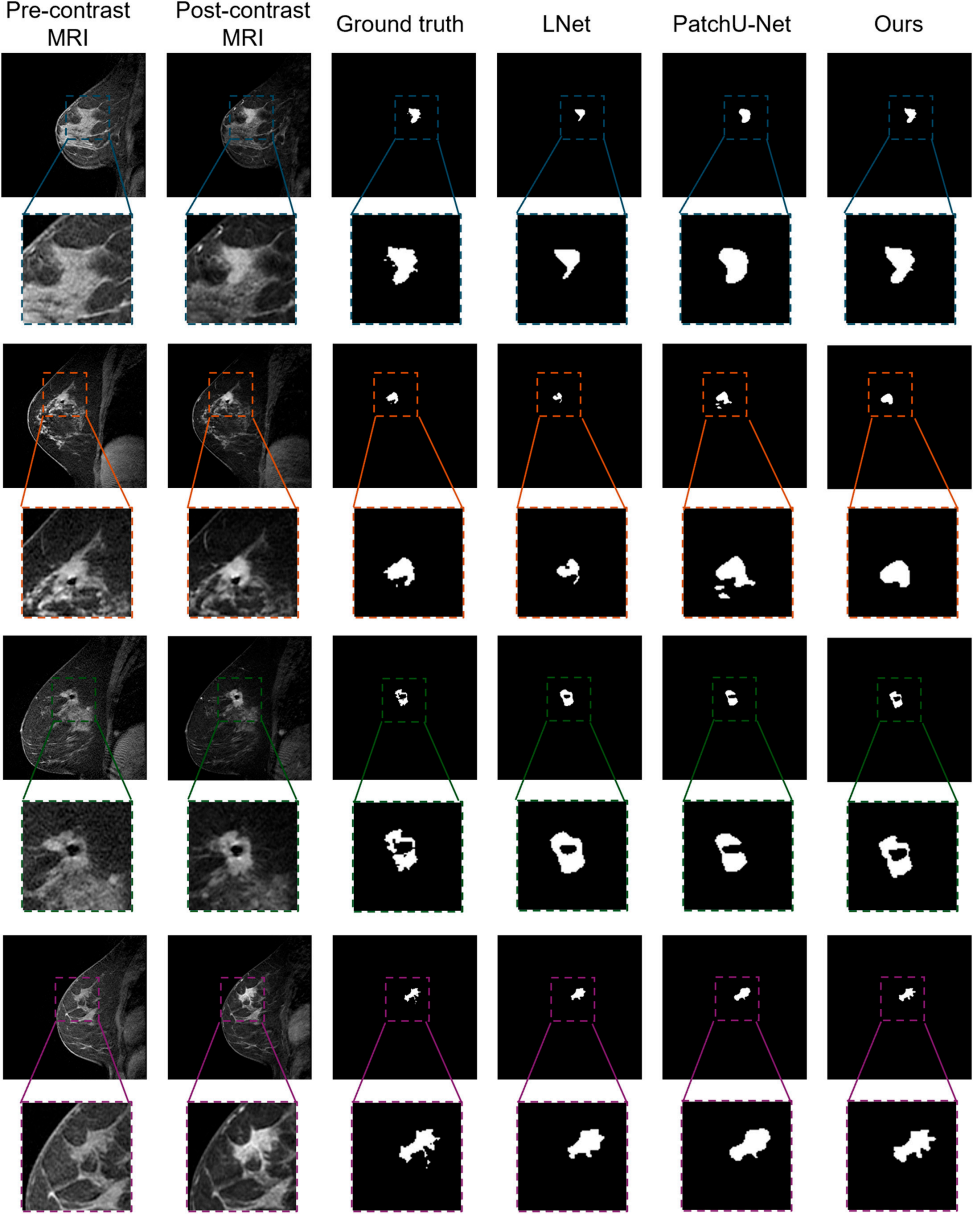
Visual comparison between existing 4D state-of-the-art approaches and our proposed 4D segmentation method in annotating breast tumors.

The qualitative assessment, illustrated in Figs. 3 and 4, visually compares the segmentation performance of various state-of-the-art methods on the TCGA-BRCA dataset. Each row represents a different patient case, displaying the pre-contrast MRI, post-contrast MRI, ground truth annotations, and segmentation results from multiple methods.

As shown in these figures, traditional U-Net and its variants (MultiResUNet, DCUNet) perform reasonably well in segmenting tumors from surrounding tissue but often fail to capture the full tumor extent, especially in cases with complex tumor morphology. In contrast, 3D-based methods such as DANet and MTLN leverage volumetric information to improve performance; however, they still exhibit instances of over-segmentation or under-segmentation. The 4D-based approaches, LNet and 3D Patch U-Net, demonstrate significant improvements by incorporating temporal dynamics. Nevertheless, our proposed method consistently achieves more accurate and detailed tumor delineation, precisely capturing intricate tumor boundaries. This superior performance likely results from the effective integration of spatiotemporal information. Overall, the qualitative comparison underscores the efficacy of our method in accurately segmenting breast tumors in DCE-MRI scans, which is crucial for precise diagnosis and treatment planning in breast cancer management.

### External validation

To further demonstrate the generalization capability of our proposed model, we conducted additional evaluations on an independent external test set collected from a different MRI center. This provided a more challenging scenario that better reflects real-world clinical variability. Quantitative results, as reported in Table 3, indicate that our approach maintains competitive performance compared to the internal test set, thereby validating its effectiveness and robustness across different institutions.

**Table 3.** 4D segmentation performance of various approaches on an external dataset (mean ± SD)

Method	Modality	DSC/%	JI/%	RVD/voxel
LNet	4D	69.3 ± 2.6	58.7 ± 2.4	1.8 ± 0.9
3D Patch U-Net	4D	70.1 ± 2.8	59.1 ± 2.5	1.7 ± 1.0
Ours	4D	73.8 ± 2.2	62.9 ± 2.0	1.6 ± 0.9

### Ablation study

To evaluate the contribution of each component in the proposed method, we conducted an ablation study by assessing different RST2G variants:

• RST2G without local information in the CFormerEncoder (w/o CFE-L): excludes convolutional layers responsible for local feature extraction.
• RST2G without global information in the CFormerEncoder (w/o CFE-G): excludes VitBlocks responsible for global feature extraction.
• RST2G without the MSR module (w/o MSR): removes the MSR module.
• RST2G without the spatiotemporal graph enhancement module (w/o STGE): removes the spatiotemporal graph enhancement module.

**Fig. 5.**
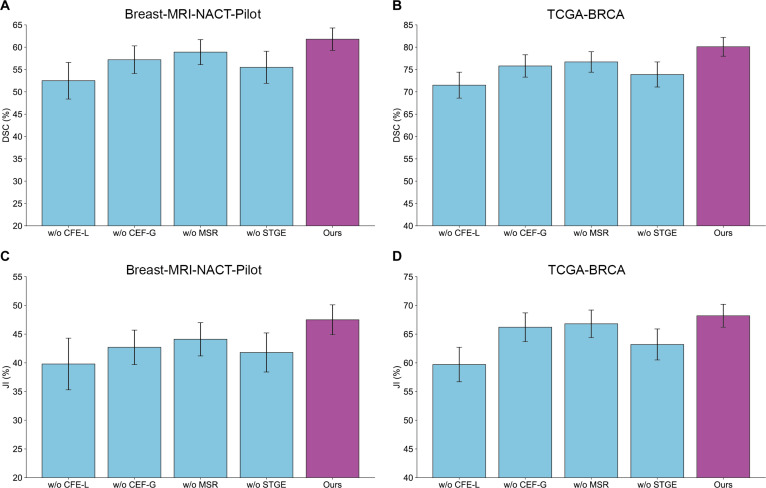
Quantitative comparison between different variants and the complete RST2G method in 2 different datasets. (A and B) Comparison of DSC metric on Breast-MRI-NACT-Pilot (A) and TCGA-BRCA (B) datasets. (C and D) Comparison of JI metric on Breast-MRI-NACT-Pilot (C) and TCGA-BRCA (D) datasets (mean ± SD).

Table 4 and Fig. 5 summarize the quantitative results of different model variants. It can be observed that all variants of RST2G obtain the decreased performances, verifying that all components can contribute to cancer segmentation. In addition, the following findings can be observed. (a) Removing the convolutional layers for local feature extraction (w/o CFE-L) causes the most significant performance drop, with the DSC decreasing from 61.8% to 52.5% on the Breast-MRI-NACT-Pilot dataset and from 80.1% to 71.5% on the TCGA-BRCA dataset. This highlights the critical role of local spatial information in accurately delineating tumor boundaries and capturing fine-grained texture details; (b) Excluding the ViT blocks that capture global contextual information (w/o CFE-G) also leads to a notable decline in segmentation accuracy, with DSC reductions of approximately 4.6% and 4.3% on the 2 datasets, respectively. This underscores the importance of modeling long-range dependencies and global spatial relationships to address tumor heterogeneity and complex shapes; (c) Removing the MSR module (w/o MSR) results in a moderate performance decrease, indicating that fusing multi-modal features and recalibrating them via spatial attention enhances feature discriminability and robustness; (d) Omitting the spatiotemporal graph enhancement module (w/o STGE) causes a significant drop in segmentation performance, confirming that explicitly modeling spatiotemporal contextual dependencies through graph-based attention is essential for capturing dynamic tumor characteristics and improving segmentation consistency across slices and time points.

**Table 4.** Ablation study on 2 public datasets

	Breast-MRI-NACT-Pilot			TCGA-BRCA		
	DSC/%	JI/%	RVD/voxel	DSC/%	JI/%	RVD/voxel
Ours	61.8 ± 2.5	47.5 ± 2.6	1.8 ± 1.1	80.1 ± 2.1	68.2 ± 2.0	1.5 ± 0.8
w/o CFE-L	52.5 ± 4.1	39.8 ± 4.5	2.9 ± 1.8	71.5 ± 2.9	59.7 ± 3.0	2.0 ± 1.3
w/o CFE-G	57.2 ± 3.1	42.7 ± 3.0	2.1 ± 1.2	75.8 ± 2.5	66.2 ± 2.5	1.7 ± 1.1
w/o MSR	58.9 ± 2.8	44.1 ± 2.9	2.0 ± 1.0	76.7 ± 2.3	66.8 ± 2.4	1.7 ± 1.0
w/o STGE	55.5 ± 3.6	41.8 ± 3.4	2.4 ± 1.3	73.9 ± 2.8	63.2 ± 2.7	1.8 ± 1.1

**Fig. 6.**
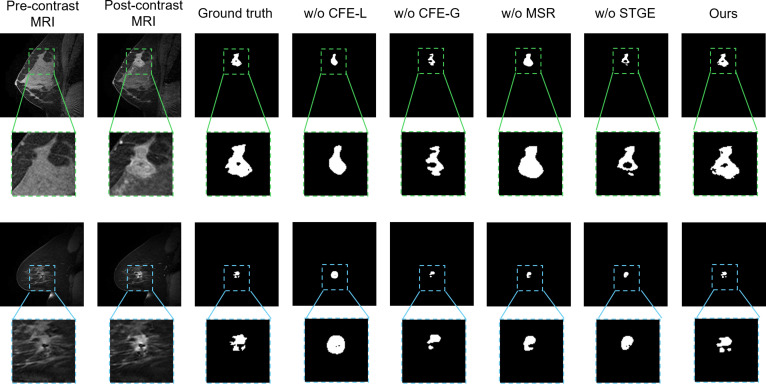
Visual comparison between different variants (w/o CFE-L, w/o CFE-G, w/o MSR, and w/o STGE) and the complete RST2G method in annotating breast tumors.

Fig. 6 provides a visual comparison of different RST2G variants against the complete method on the TCGA-BRCA dataset. The results show that removing the convolutional layers for local feature extraction (w/o CFE-L) leads to the most significant performance drop, with segmentation masks often failing to accurately capture tumor boundaries and fine details. Excluding the ViT blocks for global context (w/o CFE-G) also notably reduces accuracy, particularly in complex tumor cases. The absence of the MSR module (w/o MSR) results in less precise masks, while omitting the spatiotemporal graph enhancement module (w/o STGE) causes a marked decrease in segmentation accuracy and consistency. Overall, the complete RST2G method produces the most accurate and detailed segmentation masks, demonstrating the importance of integrating local and global information, refining multi-scale features, and enhancing spatiotemporal relationships for effective breast tumor segmentation in DCE-MRI scans.

### Hyperparameter analysis

**Fig. 7.**
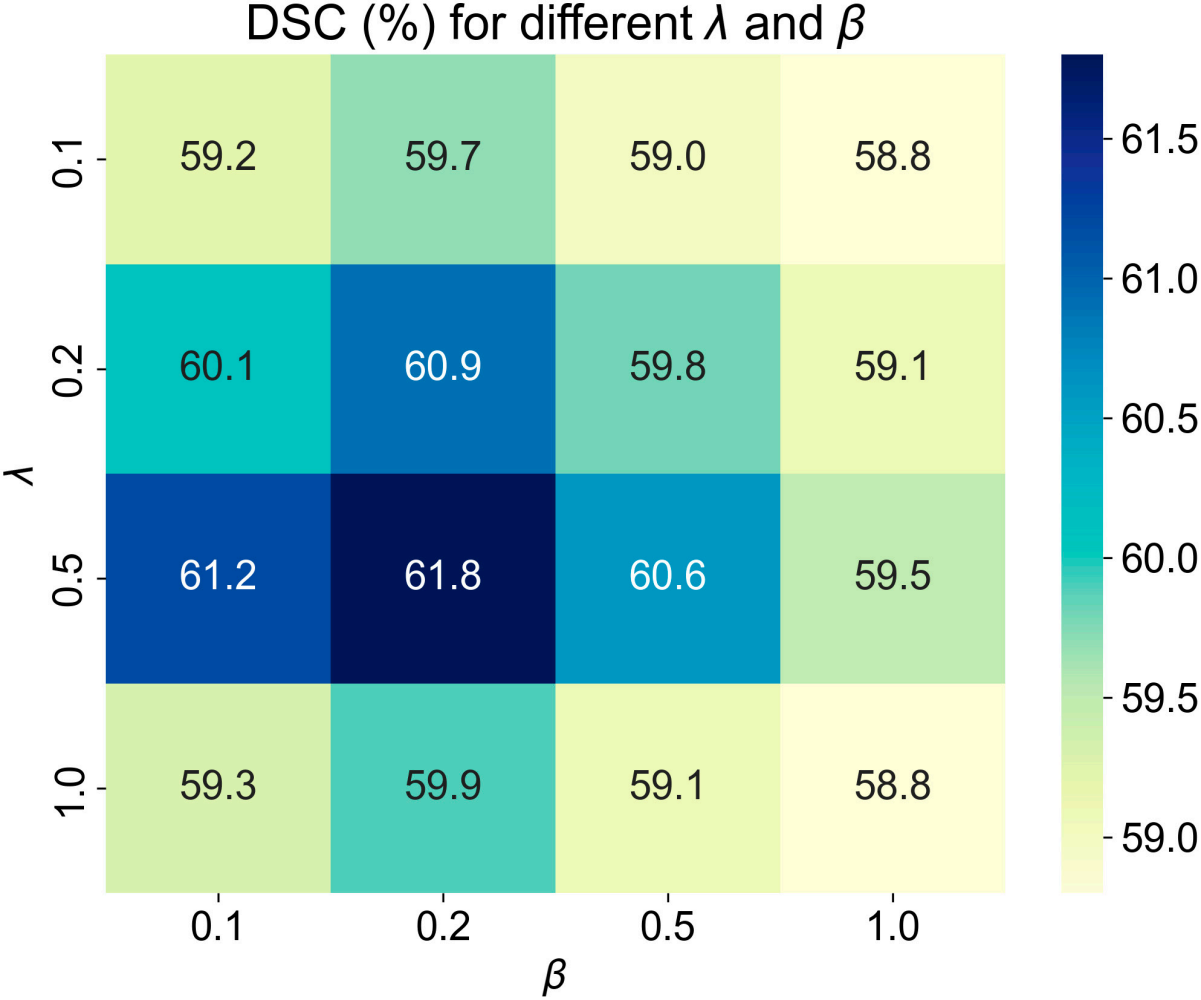
Analysis of hyperparameter sensitivity concerning the balancing coefficients λ and β that control the contribution of different objective functions.

We adjust the coefficients λ and β to investigate the contribution of different objective functions. Specifically, the values of λ and β are searched within the range of 0.1, 0.2, 0.5, and 1.0, and RST2G achieves optimal performance in terms of DSC when λ=0.5 and β=0.2. The results are summarized in Fig. 7. Thus, the hyperparameters λ and β are empirically set to 0.5 and 0.2, respectively.

### Model interpretability

**Fig. 8.**
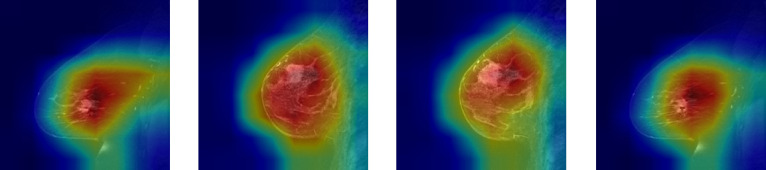
Grad-CAM visualization of the proposed RST2G model for different slices.

We applied Grad-CAM to the convolutional backbone to generate class activation maps, which highlight the discriminative regions of the input medical images that contribute most significantly to the model’s predictions. This visualization technique enables us to gain insights into the decision-making process of the deep learning model by localizing the spatial areas where the learned features are most salient. By overlaying the activation maps on the original images, we can qualitatively assess whether the model focuses on clinically relevant structures, thereby enhancing the interpretability and trustworthiness of the model in a medical context. As shown in Fig. 8, the proposed RST2G was able to effectively localize tumor regions, demonstrating its interpretability in clinical applications.

## Discussion

The experimental results presented in this study demonstrate the significant advantages of the proposed RST2G model for segmenting breast tumors from longitudinal DCE-MRI scans. By effectively integrating spatial, temporal, and residual information, our method consistently outperforms state-of-the-art 2D, 3D, and 4D segmentation approaches across 2 diverse datasets with varying imaging protocols. This superior performance can be attributed to several key aspects of our model design.

First, the explicit incorporation of residual images—representing the difference between post-contrast and pre-contrast scans—provides critical kinetic information that enhances the model’s ability to capture dynamic contrast enhancement patterns. Unlike many existing methods that implicitly model temporal changes, our residual-guided MSR module explicitly leverages these inter-temporal differences, resulting in more discriminative feature representations. Second, the hybrid architecture of the CFormerEncoder, which combines convolutional operations with transformer-based self-attention, enables effective capture of both local texture details and long-range dependencies. This capability is particularly important in breast tumor segmentation, where tumors exhibit high heterogeneity in shape, size, and internal texture. The MSR further enhances feature expressiveness by fusing complementary information from multiple modalities, thereby improving robustness against variability in tumor appearance. Third, the spatiotemporal graph enhancement module, incorporating ISA and ITA, plays a crucial role in modeling contextual relationships both within and across imaging slices and time points. This graph-based attention mechanism facilitates the integration of spatial and temporal cues, enabling more precise tumor boundary delineation and improved longitudinal consistency.

While our proposed RST2G framework demonstrates promising performance on 2 widely used public breast DCE-MRI datasets, several challenges remain for clinical integration and broader applicability. (a) Clinical integration challenges: Clinical deployment requires compatibility with diverse imaging protocols, real-time operational constraints, and seamless integration into radiologists’ workflows. Differences in image resolution, contrast agent dosing, and scanning sequences across institutions can affect model performance. Additionally, interpretability and user trust are critical for adoption, necessitating further validation and possible explainability modules; (b) Multi-center generalization: Our experiments focused on publicly available datasets with relatively homogeneous acquisition conditions. In real-world scenarios, scanner models, magnetic field strengths, and imaging parameters vary across centers. This heterogeneity can degrade segmentation accuracy if not accounted for. Future work should evaluate RST2G on multi-center datasets and may require domain adaptation techniques or augmentation strategies to enhance robustness; (c) Handling irregular temporal sampling and domain adaptation: DCE-MRI protocols often differ in the number and timing of post-contrast acquisitions, leading to irregular temporal sampling. RST2G currently assumes a fixed temporal sequence for residual computation and attention modeling. Adapting the framework to handle variable-length sequences or missing time points using techniques such as temporal alignment, imputation, or transformer models designed for irregular time series is a promising direction. Furthermore, domain adaptation approaches, including unsupervised adversarial learning or self-supervised pretraining on unannotated multi-center datasets, could mitigate domain shift and improve generalization. Addressing these aspects is crucial for bridging the gap between research and clinical practice. Future investigations will focus on rigorous multi-institutional validation, efficiency optimizations, and extensible model designs to tackle these challenges. In conclusion, the proposed RST2G framework advances breast tumor segmentation in longitudinal DCE-MRI by effectively exploiting spatial, temporal, and residual information through a novel transformer-graph architecture. Its superior quantitative and qualitative performance suggests promising potential for improving breast cancer diagnosis, treatment planning, and monitoring in clinical practice.

### Related works

#### Breast cancer segmentation in DCE-MRI

Breast cancer segmentation in DCE-MRI has garnered significant research interest due to the modality’s rich spatiotemporal information and clinical relevance. Early methods primarily relied on traditional image processing and machine learning techniques, such as region growing, level sets, and support vector machines, which depended on handcrafted features and exhibited limited generalization capabilities. With the advent of deep learning, CNNs have become the dominant approach for tumor segmentation. For example, U-Net and its variants have been widely adopted to exploit multi-scale features and enable end-to-end segmentation [30,31]. However, these methods typically focus on spatial features extracted from individual time points or aggregated volumes, often neglecting the temporal dynamics inherent in DCE-MRI sequences. To better leverage temporal information, several studies have incorporated long short-term memory (LSTM) units [32] or hemodynamic priors to model contrast enhancement patterns over time [5,29,33,34,35]. Despite these advances, existing methods primarily model dynamic contrast enhancement implicitly and often overlook the full spatiotemporal information inherent in DCE-MRI. Consequently, these approaches still face challenges in handling the high variability of tumor appearance. More recently, a handful of breast tumor segmentation methods were proposed. Huang et al. [36] utilized a joint-phase attention network for tumor segmentation in DCE-MRI. Chen et al. [37] proposed ESKNet that employed an enhanced adaptive selection convolution for breast tumor segmentation. Zhou et al. [38] designed a prototype learning-guided hybrid network for breast tumor segmentation in DCE-MRI. Although these recent models incorporate advanced attention mechanisms and hybrid architectures to better capture spatiotemporal features, they still face limitations in effectively modeling long-range dependencies across both spatial and temporal dimensions inherent in DCE-MRI data.

#### Residual learning

Residual learning, initially introduced to address gradient vanishing issues in image recognition [39], has proven highly effective by enabling models to focus on learning residuals between inputs and outputs. This approach has been successfully extended to generative tasks. For instance, Jifara et al. [40] applied residual learning in autoencoders for image denoising, enhancing the network’s ability to restore image details. Gao et al. [41] proposed a 2-level residual CNN for super-resolution imaging to capture high-frequency components. More recently, Huang et al. [42] integrated residual learning into ViTs, facilitating the training of deeper networks and mitigating degradation problems. Inspired by these advancements, we incorporate residual learning into our RST2G model. Specifically, we introduce residuals between post-contrast and pre-contrast MRI as model inputs to explicitly capture inter-temporal kinetic information. Additionally, we propose an MSR module to further enhance feature representation.

## Methods

### Overview

The proposed RST2G model segments breast cancer in DCE-MRI by effectively leveraging spatiotemporal information and residual learning. The overall architecture is illustrated in Fig. 1B. The model takes pre-contrast MRI, post-contrast MRI, and their residual images as inputs, which are processed through a weight-sharing CFormerEncoder to extract both local and global features. A residual-guided MSR module enhances the expressiveness of these features. Subsequently, pre-contrast, post-contrast, and residual graphs are constructed. Spatiotemporal graph enhancement is performed via ISA and ITA mechanisms to capture contextual information. Finally, the enhanced graph is fed into a CDecoder to generate the segmentation output. Our model inputs include pre-contrast MR images capturing baseline tissue anatomy, post-contrast MR images containing functional enhancement signals, and residual difference images calculating voxel-wise changes between pre- and post-contrast scans. The residual images emphasize dynamic contrast uptake, facilitating improved tumor delineation by accentuating regions with significant enhancement changes. This fusion aligns with clinical diagnostic practice and enhances the extraction of discriminative features for precise segmentation.

### CFormerEncoder

The CFormerEncoder comprises several hybrid DownBlocks that combine CNN layers with ViTs [43] to extract rich spatial features. This design leverages convolutional operations for local feature extraction and transformer-based self-attention for capturing long-range dependencies. We opted for a weight-sharing encoder design for the following reasons: (a) Parameter efficiency and regularization: Sharing encoder weights encourages the model to learn generalized feature representations that are invariant across imaging phases, which can help to regularize training and reduce the risk of overfitting, especially given the limited size of medical imaging datasets. (b) Encouraging cross-modal consistency: The underlying anatomical structures remain consistent across pre- and post-contrast images and their residuals. Weight sharing implicitly enforces the model to focus on common features, while residual branches emphasize changes, striking a balance between shared representation and modality-specific information. Fig. 1C details each DownBlock, which includes max pooling, convolutional layers, batch normalization, and ReLU activation. Formally, a convolutional block is defined as follows:

$$X_{1}=\text{ReLU}\left(\mathrm{BN}\left({W_{1}}^{*}X_{0}+b_{1}\right)\right)$$ (1)

where $X_{0}\in {\mathbb{R}}^{C_{\text{in}}\times H\times W}$ is the input tensor, $W_{1}$ denotes the convolutional kernel weights of size *N* × *N*, ${}^{*}$ represents the convolution operation, BN is batch normalization, and ReLU is the activation function. In each DownBlock, a residual connection is added to facilitate gradient flow and enable deeper network training. Afterward, the feature map $X\in {\mathbb{R}}^{B\times C\times H^{\prime }\times W^{\prime }}$ obtained from the DownBlocks is reshaped into a sequence of flattened patches:

$$Z_{0}=\left[z_{1},\;z_{2},\ldots ,\;z_{N}\right]\in {\mathbb{R}}^{B\times N\times D}$$ (2)

where $N=H^{\prime }\times W^{\prime }$ is the number of patches, and D=C is the embedding dimension. Fig. 1D displays the detailed structures of the VitBlock. The core of the ViT is the multi-head self-attention (MHSA) mechanism (Fig. 1E). For each attention head *h*, the queries $Q_{h}$, keys $K_{h}$, and values $V_{h}$ are computed by linear projections:

$$Q_{h}=Z_{0}W_{h}^{Q},\;K_{h}=Z_{0}W_{h}^{K},\;V_{h}=Z_{0}W_{h}^{V}$$ (3)

The scaled dot-product attention is then calculated as follows:

$$\text{Attention}\left(Q_{h},\;K_{h},\;V_{h}\right)=\text{softmax}\left(\frac{Q_{h}K_{h}^{⊤}}{\sqrt{d_{k}}}\right)V_{h}$$ (4)

where $d_{k}$ is the dimension of the queries and keys. The outputs of all heads are concatenated and linearly transformed:

$$\text{MHSA}\left(Z_{0}\right)=\text{Concat}\left({\text{head}}_{1},\ldots ,\;{\text{head}}_{H}\right)W^{O}$$ (5)

where *H* is the number of heads and $W^{O}$ is a learnable weight matrix.

### Multi-scale refinement

To further enhance the representation of the post-contrast features within the CFormerEncoder, we introduce an MSR module that effectively fuses multi-modal features extracted from each DownBlock. As shown in Fig. 1F, this module leverages complementary information from the pre-contrast features, post-contrast features, and their residual differences to produce an optimized and discriminative feature map. Given input features $Pre,\text{Post},Res\in {\mathbb{R}}^{B\times C\times H\times W}$, the MSR first applies modality-specific convolutional transformations:

$$\hat{P}re,\hat{P}ost,\hat{R}es=f_{\text{conv}}\left(Pre\right),f_{\text{conv}}\left(\text{Post}\right),f_{\text{conv}}\left(Res\right)$$ (6)

where $f_{\text{conv}}\left(\cdot \right)$ denotes a 3 × 3 convolution followed by batch normalization and ReLU activation. These features are concatenated and fused via a shared MLP applied channel-wise:

$$F_{\text{fused}}=MLP\left(\text{Concat}\left[\hat{P}re,\;\hat{P}ost,\;\hat{R}es\right]\right)\in {\mathbb{R}}^{B\times C\times H\times W}$$ (7)

Finally, a spatial attention map $A=\sigma \left({\text{Conv}}_{1\times 1}\left(F_{\text{fused}}\right)\right)$ is computed and applied to recalibrate the fused features:

$$F_{\text{out}}=F_{\text{fused}}⊙A$$ (8)

where σ is the sigmoid function, ⊙ denotes element-wise multiplication, and $F_{\text{out}}$ serves as the refined discriminative representation.

### Spatiotemporal graph enhancement

After extracting refined features for pre-contrast MRI, post-contrast MRI, and residual MRI from the CFormerEncoder, we perform spatiotemporal graph-based fusion to fully exploit contextual dependencies across slices and temporal modalities. This fusion is achieved through 2 complementary attention mechanisms: ISA and ITA.

#### Inter-slice attention

ISA captures spatial contextual relationships among adjacent slices within the same modality across a batch. Given a batch of feature maps ${\left\{F_{b}\right\}}_{b=1}^{B}$ for each modality, where $F_{b}\in {\mathbb{R}}^{C\times H\times W}$, we first apply modality-specific convolutional transformations to enhance local features: ISA aims to capture spatial dependencies among slices within a batch for each modality independently. Given a batch of feature maps $F^{\left(m\right)}\in {\mathbb{R}}^{B\times C\times H\times W}$ for modality $m\in \left\{\mathrm{pre},\;\text{post},\;d\right\}$, we first apply a convolutional transformation to enhance local features:

$${\hat{F}}^{\left(m\right)}=f_{\text{conv}}^{\left(m\right)}\left(F^{\left(m\right)}\right),\;f_{\text{conv}}^{\left(m\right)}:{\mathbb{R}}^{C\times H\times W}\to {\mathbb{R}}^{C\times H\times W}$$ (9)

Next, the spatial dimensions are flattened to form graph nodes:

$$Z^{\left(m\right)}\in {\mathbb{R}}^{B\times N\times C},\;N=H\times W$$ (10)

A graph attention layer (GAT) [44] is then applied to model pairwise relationships between nodes. The GAT computes attention coefficients $\alpha_{\mathrm{ij}}$ between nodes *i* and *j* as:

$$\alpha_{ij}=\frac{\exp\left(\text{LeakyReLU}\left(a^{⊤}\left[Wh_{i}∥Wh_{j}\right]\right)\right)}{\sum_{k=1}^{N}\exp\left(\text{LeakyReLU}\left(a^{⊤}\left[Wh_{i}∥Wh_{k}\right]\right)\right)}$$ (11)

where W is a learnable linear transformation, a is the attention vector, and ∥ denotes concatenation. The output node features are aggregated as follows:

$$h_{i}^{\text{out}}=\sigma \left(\sum_{j=1}^{N}\alpha_{ij}Wh_{j}\right)$$ (12)

Finally, the graph-enhanced features are reshaped back to spatial maps ${\hat{F}}^{\left(m\right)}\in {\mathbb{R}}^{B\times C\times H\times W}$. To further leverage inter-slice correlations within the batch, we partition the feature maps along the height dimension into *H* slices, each with shape ${\mathbb{R}}^{B\times C\times W}$.

A batch-wise attention mechanism recalibrates features across these slices. Specifically, the feature tensor is permuted and reshaped to aggregate the batch and height dimensions, resulting in a tensor of shape ${\mathbb{R}}^{\left(B\times H\right)\times C\times W}$. This arrangement treats the batch dimension as a proxy for different slices, enabling the model to capture dependencies and contextual relationships between slices. Recalibration is performed via a lightweight attention module composed of a 1 × 1 convolution followed by a sigmoid activation, which generates adaptive attention weights for each channel and spatial location. These weights are applied multiplicatively to the original features, effectively emphasizing informative features while suppressing less relevant ones. Finally, the recalibrated features are reshaped and permuted back to their original dimensions ${\mathbb{R}}^{B\times C\times H\times W}$, preserving spatial structure while enhancing inter-slice feature interactions.

#### Inter-temporal attention

After enhancing each modality’s features via ISA, we perform inter-temporal fusion to integrate complementary information across modalities. The recalibrated features ${\hat{F}}^{\left(\mathrm{pre}\right)},{\hat{F}}^{\left(\text{post}\right)},{\hat{F}}^{\left(\mathrm{res}\right)}$ are concatenated channel-wise:

$$F_{\mathrm{cat}}=\text{Concat}\left[{\hat{F}}^{\left(\mathrm{pre}\right)},\;{\hat{F}}^{\left(\text{post}\right)},\;{\hat{F}}^{\left(\mathrm{res}\right)}\right]\in {\mathbb{R}}^{B\times 3C\times H\times W}$$ (13)

An MLP is applied in a channel-wise manner to fuse these features into a unified representation:

$$F_{\text{fused}}=\mathrm{MLP}\left(F_{\mathrm{cat}}\right)\in {\mathbb{R}}^{B\times C\times H\times W}$$ (14)

To adaptively emphasize salient spatial features, a 1 × 1 convolution followed by a sigmoid activation generates an attention map:

$$A=\sigma \left({\text{Conv}}_{1\times 1}\left(F_{\text{fused}}\right)\right)$$ (15)

The final fused feature map is obtained by element-wise multiplication:

$$F_{\text{out}}=F_{\text{fused}}⊙A$$ (16)

### Objective function

To effectively train the proposed model, we employ a hybrid loss function that combines Dice loss, BCE loss, and boundary loss [45]:

$$L=L_{\text{Dice}}+\lambda L_{\mathrm{BCE}}+\beta L_{\text{Boundary}}$$ (17)

$$L_{\text{Dice}}=1-\frac{2\sum_{i}p_{i}g_{i}+\epsilon }{\sum_{i}p_{i}+\sum_{i}g_{i}+\epsilon }$$ (18)

$$L_{\mathrm{BCE}}=-\frac{1}{N}\sum_{i=1}^{N}\left[g_{i}\log\left(p_{i}\right)+\left(1-g_{i}\right)\log\left(1-p_{i}\right)\right]$$ (19)

$$L_{\text{Boundary}}=\frac{1}{N}\sum_{i=1}^{N}\left|\text{Sobel}\left(p_{i}\right)-\text{Sobel}\left(g_{i}\right)\right|$$ (20)

where $\lambda ,\beta \in \left[0,1\right]$ is a hyperparameter that balances the contribution of each loss component, $p_{i}$ and $g_{i}$ denote the predicted probability and ground truth label for voxel *i*, respectively, ϵ is a small constant added for numerical stability, Sobel operator Sobel is applied using two 3 × 3 kernels to compute the gradients in the horizontal and vertical directions, and *N* is the total number of voxels.

### Datasets

In this study, we evaluate the proposed method using 2 publicly accessible breast cancer datasets [15] that differ in their imaging protocols. The first dataset, Breast-MRI-NACT-Pilot [16], comprises longitudinal DCE-MRI scans from 64 patients undergoing NACT for invasive breast cancer. For each patient, at least 3 time points were acquired during the contrast-enhanced MRI protocol: a pre-contrast scan followed by 2 consecutive post-contrast scans. The contrast agent gadopentetate dimeglumine was administered at a dose of 0.1 mmol/kg body weight, followed by a 10-ml saline flush, with injection synchronized to the start of the early phase acquisition. The post-contrast imaging was performed at 2.5 and 7.5 min after contrast injection using standard *k*-space sampling. Each breast MR volume consists of 60 slices, each with a resolution of 256 × 256 pixels. Tumor annotations are provided in this dataset. The second dataset, TCGA-BRCA [17], contains longitudinal DCE-MRI data from 139 participants. These breast MRIs were acquired using GE 1.5 Tesla scanners (GE Medical Systems, Milwaukee, WI, USA), including one pre-contrast image and between 3 and 5 post-contrast images following contrast agent injection. The in-plane resolution ranges from 0.53 to 0.85 mm, with slice thickness varying between 2 and 3 mm. Each MR volume comprises 60 slices, each sized 256 × 256 pixels. Tumor annotations are provided in this dataset.

### Evaluation metrics

We validate the effectiveness of the proposed method using several representative metrics, including DSC, JI, and RVD. These metrics are formally defined as follows:

$$\mathrm{DSC}=\frac{2|P\cap G|}{|P|+|G|}$$ (21)

$$\mathrm{JI}=\frac{|P\cap G|}{|P\cup G|}$$ (22)

$$\mathrm{RVD}=\frac{|P|-|G|}{|G|}$$ (23)

where P denotes the predicted segmentation mask and G represents the ground truth mask. DSC and JI measure the overlap between the predicted and ground truth regions, with higher values indicating better segmentation accuracy. The RVD quantifies the relative difference in volume between the prediction and ground truth, where values closer to zero indicate more accurate volume estimation.

### Implementation details

The proposed model was developed using the PyTorch framework and trained on 2 NVIDIA Tesla V100 GPUs to accelerate computation. Optimization was performed using the Adam optimizer [19] with an initial learning rate of 10^−3^ and a weight decay of 10^−4^. The batch size was set to 4. The hyperparameters λ and β were empirically set to 0.5 and 0.2, respectively, to balance the loss components. To mitigate the risk of overfitting and to provide a more reliable assessment of our method’s performance, we employed a stratified *k*-fold cross-validation scheme (with *k* = 5) in all experiments. To ensure a fair comparison, all models, including ours and the baselines, were randomly initialized without pretraining on external datasets. The dataset was split into 70% training and validation, and 30% testing, ensuring patient-level separation to avoid data leakage. The number of transformer layers is 6, and the number of heads in the multi-head attention mechanism is 4. No data augmentation was applied during training to ensure fairness.

### Computational efficiency analysis

To evaluate the feasibility of deploying RST2G in clinical settings, we analyzed its computational complexity and runtime performance. The model size of RST2G is approximately 85.24 million parameters, indicating a moderate memory footprint suitable for practical deployment. During inference, processing a single volume takes roughly 30 s on our evaluation hardware, which corresponds to a GPU memory consumption of 10 GB. Although RST2G requires higher computational resources compared to UENTER (which has 62.88 million parameters and requires 5-GB GPU memory with 23-s inference time), the increase in resource usage is justified by the improvement in model capacity and performance. These results demonstrate that RST2G achieves a favorable balance between accuracy and computational efficiency, supporting its potential for real-time or near-real-time clinical applications.

### Competing methods

To evaluate the effectiveness of the proposed method, we compared it against several state-of-the-art segmentation approaches spanning 2D, 3D, and 4D frameworks. The 2D methods include U-Net [20], MultiResUNet [21], DCUNet [22], and TEBLS [23], while the 3D methods comprise V-Net [24], DANet [25], MTLN [26], and 3DUNETER [27]. The 4D approaches evaluated are LNet [28] and 3D Patch U-Net [29]. Specifically, U-Net is a widely adopted encoder–decoder architecture with skip connections that facilitate precise localization and context integration. MultiResUNet enhances U-Net by incorporating multi-resolution analysis to capture features at different scales, whereas DCUNet introduces densely connected convolutional blocks to improve feature propagation and gradient flow. V-Net extends U-Net to volumetric data using 3D convolutions for effective volumetric segmentation. DANet integrates dual attention mechanisms to capture spatial and channel-wise dependencies, improving segmentation accuracy. MTLN employs multi-task learning to jointly optimize related tasks, enhancing robustness. LNet and 3D Patch U-Net exploit temporal and spatial information in 4D data, with LNet emphasizing longitudinal consistency and 3D Patch U-Net utilizing patch-based processing to capture local details effectively.

## Data Availability

The datasets used in this study were publicly available at: https://www.cancerimagingarchive.net/collection/breast-mri-nact-pilot/ and https://www.cancerimagingarchive.net/collection/tcga-brca/. The source code was publicly available at: https://github.com/ttt553/RST2G.
